# Ascitic Interleukin 6 Is Associated with Poor Outcome and Spontaneous Bacterial Peritonitis: A Validation in Critically Ill Patients with Decompensated Cirrhosis

**DOI:** 10.3390/jcm9092865

**Published:** 2020-09-04

**Authors:** Ulrich Mayr, Marina Lukas, Mayada Elnegouly, Christine Vogt, Ulrike Bauer, Joerg Ulrich, Roland M. Schmid, Wolfgang Huber, Tobias Lahmer

**Affiliations:** 1 Klinik und Poliklinik für Innere Medizin II, Klinikum rechts der Isar, Technische Universität München, D-81675 München, Germany; marina.lukas@mri.tum.de (M.L.); mayada.elnegouly@mri.tum.de (M.E.); ulrike.bauer@mri.tum.de (U.B.); joerg.ulrich@mri.tum.de (J.U.); rolandM.schmid@mri.tum.de (R.M.S.); wolfgang.huber@mri.tum.de (W.H.); tobias.lahmer@mri.tum.de (T.L.); 2 Institut für Klinische Chemie und Pathobiochemie, Klinikum rechts der Isar, Technische Universität München, D-81675 München, Germany; christine.vogt@mri.tum.de

**Keywords:** liver cirrhosis, acute-on-chronic liver failure, biomarkers, interleukin 6, spontaneous bacterial peritonitis, intensive care unit

## Abstract

Decompensated cirrhosis predisposes to infectious diseases and acute-on-chronic liver failure (ACLF) in critically ill patients. Infections like spontaneous bacterial peritonitis (SBP) are frequently associated with multi-organ failure and increased mortality. Consequently, reliable predictors of outcome and early diagnostic markers of infection are needed to improve individualized therapy. This study evaluates the prognostic role of ascitic interleukin 6 in 64 patients with cirrhosis admitted to our intensive care unit (ICU). In addition, we analysed the diagnostic ability of ascitic interleukin 6 in a subgroup of 19 patients with SBP. Baseline ascitic interleukin 6 performed well in predicting 3-month mortality in patients with decompensated cirrhosis (area under curve (AUC) = 0.802), as well as in patients fulfilling ACLF-criteria (AUC = 0.807). Ascitic interleukin 6 showed a moderate prognostic advantage compared with common clinical scores and proinflammatory parameters. Moreover, ascitic interleukin 6 had a sufficient diagnostic ability to detect SBP (AUC = 0.901) and was well correlated with ascitic polymorphonuclear neutrophils in SBP (*p* = 0.002). Interestingly, ascitic interleukin 6 revealed a high predictive value to rule out apparent infections on admission to ICU (AUC = 0.904) and to identify patients with “culture-positive SBP” (AUC = 0.856). Ascitic interleukin 6 is an easily-applicable proinflammatory biomarker with high prognostic and diagnostic relevance in critically ill patients with liver cirrhosis.

## 1. Introduction

Liver cirrhosis is a highly-prevalent and challenging disorder with poor outcome, particularly in critically ill patients [1,2]. Impairment of hepatocellular function, portal hypertension, and ascites with increased intraabdominal pressure are typical hallmarks of decompensation [3,4]. Advanced cirrhosis is frequently complicated by acute-on-chronic liver failure (ACLF), demanding transfer to an intensive care unit (ICU) [5]. ACLF is characterized by sequential multi-organ dysfunction and dramatically high mortality rates [6]. Bacterial infections are major precipitating factors of ACLF and early identification is of vital importance for individualized treatment [7].

Cirrhosis typically predisposes to bacterial infections and septic multi-organ failure. The most prevalent are pneumonia, urinary tract infections, bacteremia, and spontaneous bacterial peritonitis (SBP) [8,9,10]. The high incidence of infections is attributed to a complex and multifactorial syndrome of cirrhosis associated immune dysfunction (CAID), leading to “immune paralysis” with insufficient host response to infectious pathogens [11,12]. Moreover, portal hypertension is accompanied by a disturbance of intestinal microcirculation with bacterial overgrowth and impairment of mucosal integrity [13]. Bacterial translocation (BT) plays a crucial role in development and aggravation of infections in cirrhotic individuals [14]. Previous studies described an association of BT with specific surrogate parameters in patients with advanced liver disease [15]. For example, the acute-phase proteins C-reactive protein (CRP) and procalcitonin (PCT) are commonly evaluated “biomarkers” for identification of infectious diseases and prediction of prognosis in patients with liver cirrhosis [16,17,18]. Interleukin 6 (IL-6) is a pleiotropic proinflammatory cytokine involved in the initiation and regulation of inflammatory responses [19,20]. IL-6 reacts faster to infectious stimuli than CRP or PCT. Its serum levels are linked to severity of inflammation and organ dysfunction [21,22,23]. Concerning levels of IL-6 in ascites, former studies described a correlation of ascitic IL-6 with the diagnosis of SBP [24,25].

SBP is defined as a bacterial infection with polymorphonuclear neutrophils (PMN) ≥ 250/µL in the ascitic fluid. Its prevalence ranges from 10 to 30% in hospitalised patients with decompensated cirrhosis [26]. Although in-hospital mortality could be reduced with early detection and therapy, SBP carries a high risk for decompensation and ACLF with renal impairment or multi-organ failure [27,28].

Consequently, the establishment of early and reliable predictors of mortality is an attractive goal, particularly in critically ill patients with cirrhosis or ACLF. Laboratory markers with high potential for timely diagnosis as well as response to treatment could further improve prognosis of bacterial infections. So far, no single biomarker could be established for both sufficient prediction of outcome as well as valid detection of manifest SBP. The aim of the present study was to evaluate the prognostic and diagnostic potential of ascitic IL-6 in a challenging population of critically ill patients with decompensated liver cirrhosis.

## 2. Materials and Methods

### 2.1. Study Design

This study was approved by the institutional review board (Ethikkommission Technische Universität München; Fakultät für Medizin; Project number 413/19s). Due to its retro-prospective nature informed consent was not feasible. We screened a total of 110 critically ill Caucasian patients with liver cirrhosis admitted to our ten-bed, university hospital medical ICU between January 2017 and April 2019. Diagnosis of cirrhosis was based on one or more of the following features: radiological, sonographic, or histological hallmarks of cirrhosis; laboratory dysfunction with impaired liver synthesis in presence of risk factors for cirrhosis; or former medical reports suggesting end-stage liver disease (i.e., variceal bleeding episodes, ascites, or hepatic encephalopathy).

We excluded all patients without puncturable ascites on admission to ICU (*n* = 9). Owing to obvious influences on laboratory and microbiological analyses, all patients with pre-existing broad-spectrum antibiotic therapy during the week before ICU admission were excluded (*n* = 16). We excluded patients who underwent liver transplantation during the observation period (*n* = 2) and patients with additional hepatocellular carcinoma (*n* = 2) or hemorrhagic ascites (*n* = 2). Patients with incomplete laboratory or microbiological analyses or insufficient exclusion of underlying infectious diseases were excluded (*n* = 12). One patient was excluded because of secondary peritonitis with proof of sigmoid perforation via CT scan. All included patients were followed up until death or a minimum observation period of 3 months; patients lost during this time period were excluded (*n* = 2). None of the included patients with SBP had pre-existing antibiotic prophylaxis with norfloxacin or rifaximin.

### 2.2. Laboratory Analyses and Clinical Scores

Blood samples were acquired irrespective of this study corresponding to current standard in our ICU. We focused on analyses of the inflammatory parameters CRP, PCT, and serum interleukin 6 (IL-6_serum_). Time of laboratory analysis was 60–120 min. Laboratory tests were used for calculation of scores defining health status like acute and physiology chronic health evaluation II (APACHE-II) or sequential organ failure assessment (SOFA). Parameters of liver function served for staging of cirrhosis in terms of Child–Turcotte–Pugh (CTP) and model of end-stage liver disease (MELD) score. Definition of ACLF was based on recommendations from the European Association for the Study of the Liver–Chronic Liver Failure (EASL-CLIF) Consortium [7], differentiating between no ACLF and ACLF-grade I–III. Parameters of organ function including creatinine, international normalized ratio (INR), white blood cell count (WBC), hepatic encephalopathy (HE), use of vasopressors, mean arterial pressure (MAP), arterial partial pressure of oxygen (P_a_O_2_), fraction of inspired oxygen (F_i_O_2_), or need for mechanical ventilation were included in the calculation of CLIF organ failure score (CLIF-OF) and CLIF acute-on-chronic liver failure score (CLIF-ACLF) [6,7,28].

Paracentesis was done on admission to ICU irrespective of the study by the treating ICU physician. It was performed ultrasound guided in a supine position and postinterventional substitution of albumin followed current guidelines [29,30]. Ascites were sent immediately for laboratory analyses of ascitic interleukin 6 (IL-6_ascites_) and PMN. Time of analysis was 60–120 min for ascitic parameters. SBP was diagnosed in all patients with PMN ≥ 250/µL [26]. Analysis of IL-6_serum_ and IL-6_ascites_ was done using an electrochemiluminescence immunoassay (ECLIA) with a detection limit of 1.5 pg/mL (Cobas 8000^®^, Roche, Basel, Switzerland); the costs were seven times as much as for PMN. All laboratory tests were realised by the department of clinical laboratory chemistry of our university hospital.

### 2.3. Microbiological Analyses and Evidence of Infection

Specimens were cultured using conventional culture techniques performed by the department of microbiology of our university hospital (time of analysis 2–3 days). Ascites were inoculated into anaerobic and aerobic blood culture bottles immediately after paracentesis. PMN ≥ 250/µL was diagnostic for SBP, regardless of the cultural isolation of bacteria. Depending on PMN, the population was divided into *“SBP*” and “*no SBP*”. In this study, we further distinguished between “*culture-positive SBP*” and patients with “*culture-negative neutrocytic ascites*” (CNNA) [31].

Routine screening for infectious diseases was done in all patients with decompensated liver cirrhosis on admission to ICU. We performed a sampling of two pairs of blood cultures to exclude bacteremia. Urine dipstick/sediment and cultures were screened for urinary tract infection. Radiological examinations via X-ray chest or CT scan served to diagnose or exclude respiratory infections. Furthermore, broncho-alveolar lavage (BAL) was performed in all patients with mechanical ventilation to diagnose specific pathogens of broncho-pulmonary infections.

These screening tests allowed a further categorization of our population. Bacteremia was confirmed if at least one blood culture was positive. Urinary tract infection was diagnosed in the case of either pathological dipstick/sediment or positive urine culture. Patients with either radiological signs of pneumonic infiltrate or evidence of specific pathogens via BAL were diagnosed as having respiratory infection. Thus, patients with at least one pathological finding in the mentioned criteria were classified as having *evidence of infection (“infection”)*. As opposed to this, patients with *no evidence of infection* on admission to ICU were categorized as “*no infection*”.

### 2.4. Data Collection

Clinical and laboratory parameters for the calculation of APACHE II score, SOFA score, MELD score, CTP score, ACLF grade, CLIF-OF, and CLIF-ACLF were recorded on the day of paracentesis and admission to ICU. Analogously, laboratory analyses of blood and ascitic parameters as well as microbiological examinations and screening for infectious diseases were done on admission to ICU. For survival analyses, patients were followed up until death or a minimum observation period of 3 months.

### 2.5. Statistical Analysis and Primary Endpoint

Continuous variables are depicted as median and interquartile range (IQR). Categorical variables are shown as percentages. To compare patient cohorts, we used the nonparametric, two-tailed Mann–Whitney test. Correlations were done using Spearman’s coefficient r_s_ and linear regressions using the coefficient R^2^. Receiver-operating-characteristic curves (ROC) were used to express the potential of IL-6_ascites_ and other variables for prediction of 3-month mortality, “SBP”, “culture-positive SBP”, and “no evidence of infection” via area under curve (AUC). Appropriate cut-offs were identified by the highest combined sensitivity and specificity using Youden’s index. In addition, we calculated the positive predictive value (PPV) and negative predictive value (NPV) to further specify the prognostic and diagnostic ability of IL-6_ascites_. Survival analyses were performed according to the Kaplan–Meier method, and all deaths were recorded as events. Comparison of survival curves was done via log-rank (Mantel–Cox) test. Associations of variables with mortality risk were calculated as hazard ratio (HR) by Mantel–Haenszel. Significance was assumed at a *p*-value < 0.05. All analyses and graphs were generated using Prism 8 (GraphPad Software, San Diego, CA, USA).

## 3. Results

### 3.1. Patients’ Characteristics and Laboratory Analyses

A total of 64 critically ill patients with decompensated liver cirrhosis were included; their characteristics on admission to ICU are presented in Table 1. A large proportion of 86% (*n* = 55) fulfilled criteria of ACLF on admission to ICU.

### 3.2. Levels of Ascitic Interleukin 6 in Infectious Diseases

The percentage distribution of different kinds of infectious diseases with corresponding median levels of IL-6_ascites_ on admission to ICU is shown in Table 2.

Approximately 83% of our study population were classified as having “*infection*” (*n* = 53), while only 17% had no evidence of infection (*n* = 17). The “*infection*” group showed much higher IL-6_ascites_ than the “*no infection*” group (*p* < 0.001).

According to PMN, a proportion of 30% were categorized as “*SBP*” (*n* = 19), whereas 30% were “*no SBP*” group (*n* = 45). The “*SBP*” group had significantly higher baseline levels of IL-6_ascites_ than the “*no SBP*” group (*p* < 0.001). A majority of 13 patients were categorized as *nosocomial SBP* with diagnosis more than 48 h after hospitalization. By contrast, *community-acquired SBP* diagnosed within 48 h and without hospitalization in the preceding 6 months was found in a minority of six patients with SBP. There was no statistically significant difference in baseline IL-6_ascites_ between *nosocomial SBP* and *community-acquired SBP* (*p* = 0.323).

Concerning patients with single-infections, SBP revealed significantly higher IL-6_ascites_ than patients with infectious diseases of respiratory (*p* = 0.007) or urinary origin (*p* < 0.001). Pneumonia was associated with higher IL-6_ascites_ than urinary tract infection, but the result was not statistically significant (*p* = 0.055). Regarding patients with SBP, the presence of co-infections was not associated with higher IL-6_ascites_ in comparison with SBP as a single infection (*p* = 0.924).

About 53% of SBP-patients had positive ascitic bacterial cultures (*n* = 10), while a little less than half had CNNA (*n* = 9). “*Culture-positive SBP*” was associated with higher IL-6_ascites_ than CNNA (*p* = 0.008). In patients with “*culture-positive SBP*”, we isolated gram-negative Escherichia coli (*n* = 3) and Enterobacter aerogenes (*n* = 1), as well as gram-positive Enterococcus faecium (*n* = 4), Enterococcus faecalis (*n* = 1), and Staphylococcus aureus (*n* = 1). We found multi-drug resistant (MDR) bacteria in 40% with Eschericia coli resistant to aminopenicillins/third-generation-cephalosporins/fluoroquinolons (*n* = 2) and Enterococcus faecium resistant to vancomycin (*n* = 2). MDR was detectable mainly in patients with *nosocomial-SBP* (*n* = 3). We identified MDR-Eschericia coli in one patient classified as *community-acquired SBP*. There were neither patients with fungal peritonitis nor patients with culture-positive ascites in the case of PMN < 250/µL in our study population.

### 3.3. Prognostic Accuracy of Ascitic Interleukin 6

**Figure 1:**
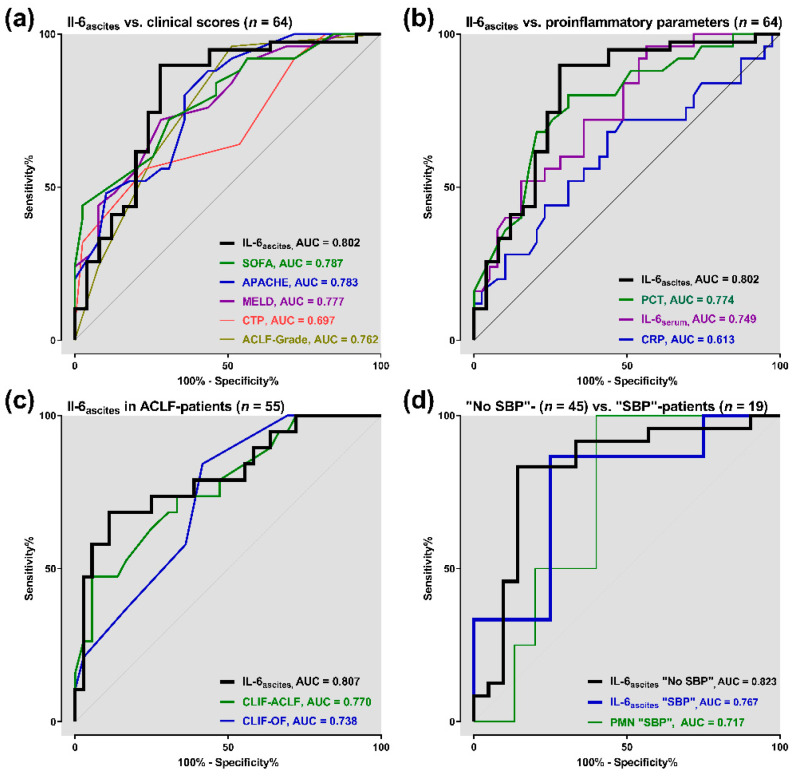
Prognostic accuracy of baseline ascitic interleukin 6 (IL-6_ascites_) to predict 3-month mortality: (**a**) IL-6_ascites_ versus clinical scores (*n* = 64); (**b**) IL-6_ascites_ versus proinflammatory parameters (*n* = 64); (**c**) IL-6_ascites_ in acute-on-chronic liver failure (ACLF) patients (*n* = 55); (**d**) IL-6_ascites_ in “*non-spontaneous bacterial peritonitis (No SBP)*” patients (*n* = 45) versus IL-6_ascites_ and polymorphonuclear neutrophils (PMN) in “*spontaneous bacterial peritonitis (SBP)*” patients (*n* = 19). APACHE: acute physiology and chronic health evaluation II; SOFA: sequential organ failure assessment; MELD: model of end-stage liver disease; CTP: Child–Turcotte–Pugh; CLIF-OF: chronic liver failure organ failure score; CLIF-ACLF: chronic liver failure acute-on-chronic liver failure score; CRP: C-reactive protein; PCT: procalcitonin; AUC: area under curve.

For primary outcome analysis, we used ROC curves to value baseline IL-6_ascites_ in prediction of 3-months mortality. In 64 ICU patients with cirrhosis, IL-6_ascites_ showed higher discriminative ability to predict the outcome (AUC = 0.802, 95% confidence interval (CI) 0.683–0.921, *p* < 0.001) than SOFA (AUC = 0.787), APACHE-II (AUC = 0.783), MELD (AUC = 0.777), CTP (AUC = 0.697), and ACLF-grade (AUC = 0.762) (Figure 1a). IL-6_ascites_ had a PPV of 79.6% and an NPV of 80% to predict mortality.

Moreover, the prognostic value of IL-6_ascites_ was better compared with conventional proinflammatory parameters PCT (AUC = 0.774), IL-6_serum_ (AUC = 0.749), and CRP (AUC = 0.613) (Figure 1b).

In 55 patients fulfilling ACLF criteria on admission to ICU, IL-6_ascites_ revealed a prognostic advantage (AUC = 0.807, 95% CI 0.681–0.934, *p* < 0.001) in comparison with CLIF-ACLF (AUC = 0.770) and CLIF-OF (AUC = 0.738) (Figure 1c).

In 45 patients of the “*no SBP”* subgroup, we found the highest potential for IL-6_ascites_ to predict 3-month mortality (AUC = 0.823). As opposed to this, prognostic value of IL-6_ascites_ was lower in 19 “*SBP*” patients (AUC = 0.767), but still better than that of PMN (AUC = 0.717, Figure 1d).

In addition, we evaluated the prognostic role of IL-6_ascites_ depending on the etiology of cirrhosis. In 48 patients with alcoholic liver cirrhosis, IL-6_ascites_ showed a higher prognostic accuracy compared with 10 patients with cryptogenic/NASH-cirrhosis (AUC = 0.814 vs. AUC = 0.760). Owing to the very limited number of patients, subgroup analyses are not available for viral-related cirrhosis (*n* = 4) or autoimmune etiology (*n* = 2).

**Figure 2:**
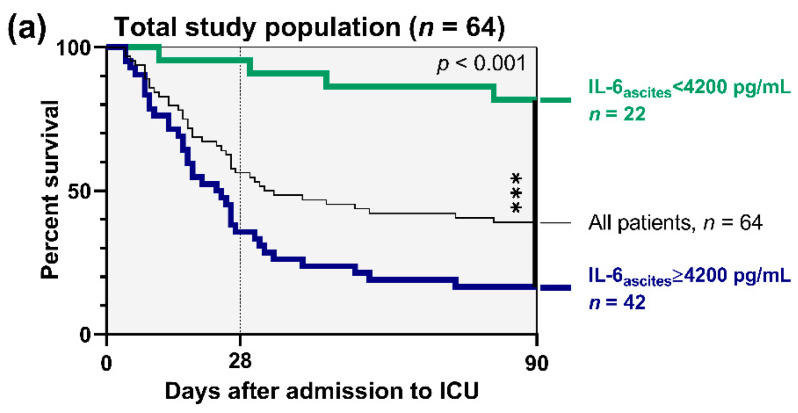

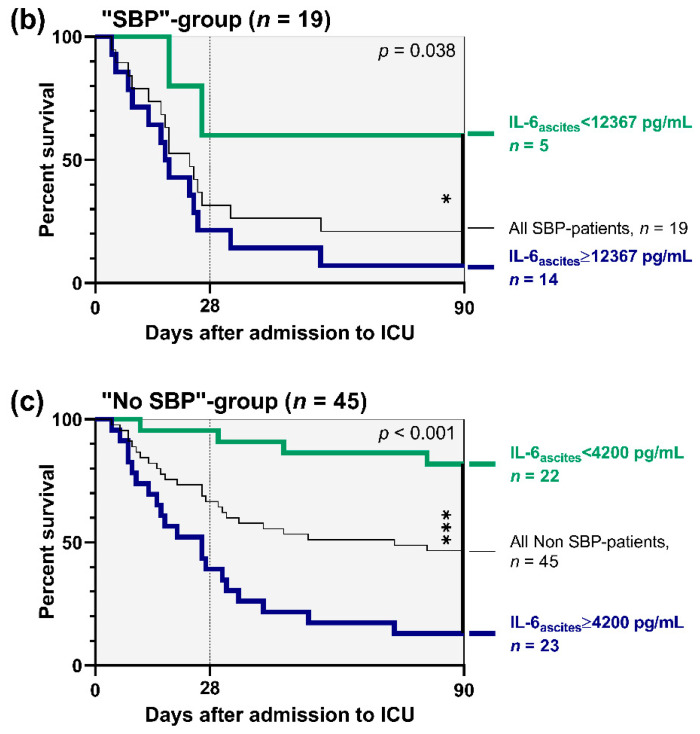
Survival analyses depending on baseline ascitic interleukin 6 (IL-6_ascites_): (**a**) total population of 64 patients (admission levels of IL-6_ascites_ < 4200 pg/mL vs. ≥ 4200 pg/mL); (**b**) “*SBP*” subgroup of 19 patients (admission levels of IL-6_ascites_ < 12,367 pg/mL vs. ≥ 12,367 pg/mL); (**c**) “*no SBP*” subgroup of 45 patients (admission levels of IL-6_ascites_ < 4200 pg/mL vs. ≥ 4200 pg/mL). ICU, intensive care unit. * = *p* < 0.05; *** = *p* < 0.001.

On the basis of these results, survival analyses were performed depending on admission levels of IL-6_ascites_. In the total population of 64 patients, we found a sensitivity of 89.7% and a specificity of 72% to predict 3-month mortality with a cut-off for IL-6_ascites_ ≥ 4200 pg/mL. As depicted in Figure 2a, admission levels of IL-6_ascites_ ≥ 4200 pg/mL were associated with significantly increased mortality risk compared with admission levels < 4200 pg/mL (hazard ratio (HR) 5.21, 95% CI = 2.73–9.97, *p* < 0.001).

Subgroup analysis in 19 individuals with “*SBP*” resulted in a sensitivity of 86.7% and a specificity of 75% to predict 3-month mortality with a cut-off for IL-6_ascites_ ≥ 12,367 pg/mL. Consequently, admission levels of IL-6_ascites_ ≥ 12,367 pg/mL in *“SBP”* patients were associated with increased 3-month mortality risk compared with admission levels < 12,367 pg/mL (HR 3.29, 95% CI = 1.14–9.50, *p* = 0.038, Figure 2b).

Analogously, a further analysis in 45 “*no SBP*” patients revealed a sensitivity of 83.3% and a specificity of 85.7% to predict mortality with a cut-off for IL-6_ascites_ ≥ 4200 pg/mL. Figure 2c illustrates that 3-month mortality risk was markedly increased in “*no SBP*” patients with admission levels of IL-6_ascites_ ≥ 4200 pg/mL compared with admission levels < 4200 pg/mL (HR 9.04, 95% CI = 3.82–21.35, *p* < 0.001).

### 3.4. Correlation Analyses

Correlation analyses and linear regressions of baseline IL-6_ascites_ with various proinflammatory parameters and clinical scores are presented in Table 3.

Subgroup analysis in 19 “*SBP*” patients resulted in a significant correlation of IL-6_ascites_ with admission levels of PMN (r_s_ = 0.661, R^2^ = 0.689, *p* = 0.002, Figure S1).

### 3.5. Diagnostic Accuracy of Ascitic Interleukin 6

**Figure 3:**
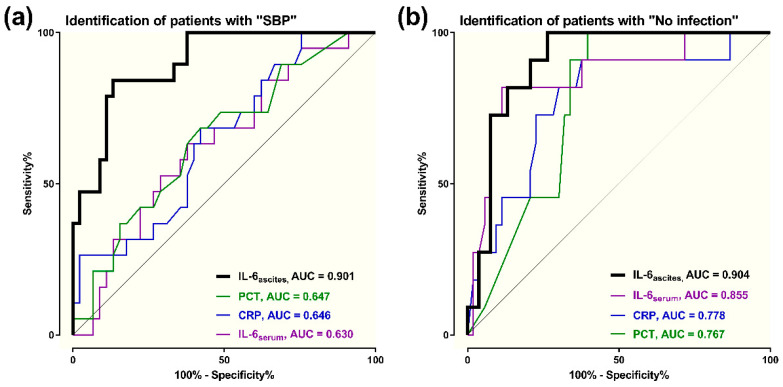
Diagnostic accuracy of baseline ascitic interleukin 6 (IL-6_ascites_), CRP, PCT, and IL-6_serum_ to (**a**) identify patients with spontaneous bacterial peritonitis (SBP) and (**b**) rule out clinically apparent infections (“*no infection*”).

ROC analyses were performed to assess the potential of IL-6_ascites_ to identify SBP with PMN ≥ 250/µL in ICU patients with liver cirrhosis. As depicted in Figure 3a, we found a sensitivity of 84.2% and a specificity of 86.7% (AUC = 0.901), with a cut-off for IL-6_ascites_ ≥ 10,037 pg/mL. Our analyses resulted in a PPV of 69.2% and an NPV of 80.4%. IL-6_ascites_ performed markedly better in detecting SBP on admission to ICU compared with PCT (AUC = 0.647), CRP (AUC = 0.646), or IL-6_serum_ (AUC = 0.630).

Further ROC-analyses were added to evaluate the discriminative ability of IL-6_ascites_ in critically ill patients with cirrhosis. Figure 3b illustrates the diagnostic value to rule out clinically apparent infections on admission to ICU: IL-6_ascites_ had a sensitivity of 81.8% and a specificity of 86.8% (AUC = 0.904), with a cut-off < 1719 pg/mL. We found a PPV of 63.6% and an NPV of 92.5%. IL-6_ascites_ performed better in identifying “*no infection*” than IL-6_serum_ (AUC = 0.855), CRP (AUC = 0.778), or PCT (AUC = 0.767).

**Figure 4:**
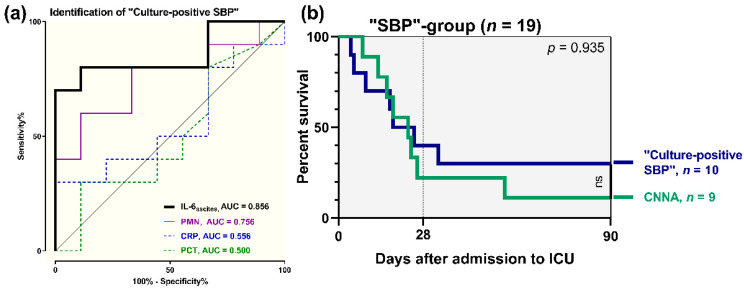
(**a**) Diagnostic potential of baseline ascitic interleukin 6 (IL-6_ascites_), polymorphonuclear neutrophils (PMN), CRP, and PCT to identify positive ascitic bacterial culture (“*culture-positive SBP*”); (**b**) survival analyses in 19 patients with spontaneous bacterial peritonitis (SBP): “*culture-positive SBP*” vs. “*culture-negative neutrocytic ascites*” (CNNA).

Concerning patients with SBP (*n* = 19), we validated the potential of IL-6_ascites_ to differentiate between “*culture-positive SBP*” and CNNA. We found a sensitivity of 80% and a specificity of 88.9% to identify “*culture-positive SBP*” (AUC = 0.856), with a cut-off for IL-6_ascites_ ≥ 28,842 pg/mL. PPV was 88.9% and NPV was 80%. In comparison, PMN performed worse in detection of “*culture positive SBP*” (AUC = 0.756), while CRP (AUC = 0.556) and PCT (AUC = 0.500) had poor discriminative ability (Figure 4a). However, according to survival analysis, “*culture-positive SBP*” on admission to ICU was not associated with a higher mortality risk in comparison with CNNA (HR 0.76, 95% CI = 0.27–2.11, *p* = 0.935) (Figure 4b).

### 3.6. Association of Ascitic Interleukin 6 with Renal Impairment

We found a significant correlation of IL-6_ascites_ with baseline creatinine on admission to ICU (r = 0.390, R^2^ = 0.065, *p* = 0.001, Figure S2).

Nevertheless, according to ROC curves, IL-6_ascites_ had a poor predictive value for identification of patients with need for hemodialysis therapy during ICU stay (AUC = 0.680). Concerning our population of critically ill patients with end-stage liver disease, SOFA (AUC = 0.825), APACHE-II score (AUC = 0.824), MELD score (AUC = 0.817), ACLF-grade (AUC = 0.766), and even CTP (AUC = 0.722) showed higher potential in predicting necessity for hemodialysis (Figure S3).

## 4. Discussion

This study primarily demonstrates the prognostic accuracy of ascitic interleukin 6 (IL-6_ascites_) in critically ill patients with cirrhosis and acute-on-chronic liver failure (ACLF).

In detail, baseline IL-6_ascites_ on admission to ICU was a prognostic factor for outcome of patients with decompensated cirrhosis; IL-6_ascites_ was moderately superior to clinical scores of health status and hepatic impairment. In a large proportion of our study population fulfilling criteria of ACLF, IL-6_ascites_ showed a slightly higher prognostic ability compared with scoring models defined by the EASL CLIF Consortium [6,7,28]. In addition, IL-6_ascites_ performed better than common proinflammatory parameters in predicting 3-month mortality. Many previous studies addressed the usefulness of CRP and PCT as predictors of mortality in cirrhosis [16,17,18,32,33], whereas data about the prognostic role of IL-6_ascites_ are rare [25,34]. Our analyses revealed a higher prognostic value of IL-6_ascites_ in comparison with CRP and PCT. Considering the high mortality of cirrhosis and ACLF with the need for enormous medical efforts, prognostic “biomarkers” might play an important role in individualized targeted therapy.

Moreover, the present study underlines the diagnostic relevance of IL-6_ascites_ as a “biomarker” for infectious diseases like SBP. We found a high correlation of IL-6_ascites_ with polymorphonuclear cell count (PMN) in patients with SBP. ROC curves revealed a sensitivity of 84.2% and a specificity of 86.7% for detection of SBP. Our results are in line with previous analyses describing higher levels of IL-6_ascites_ in the presence of SBP compared with sterile ascites [25,35]. Nieto et al. already reported a high correlation of IL-6_ascites_ with ascitic neutrophils [36]. Viallon et al. found a sensitivity of 100% and a specificity of 88% for IL-6_ascites_ to identify SBP [24]. However, none of these studies were performed in critically ill patients with cirrhosis, a population characterized by advanced illness and a high incidence of infectious diseases.

It is worth mentioning that IL-6_ascites_ performed better in diagnosing SBP than proinflammatory parameters CRP, PCT, or IL-6_serum_. This finding is contradictory to former results describing a higher diagnostic value for PCT compared with IL-6_ascites_ [24]. Various previous studies evaluated the diagnostic role of PCT, supporting its use as a “biomarker” for SBP [24,37,38,39]. Looking for the most suitable marker of SBP, multiple studies analysed the potential of other parameters such as laktoferrin [40,41], calprotectin [39,42], or leukocyte esterase activity [43]. However, the usefulness of these markers for clinical routine is restricted owing to limited availability of diagnostic assay kits [44] and often time-consuming analytics. Considering that assessment of IL-6_ascites_ is comparably quickly available as “gold-standard” PMN, IL-6_ascites_ seems to be a helpful tool for early identification of SBP and initiation of guided therapy.

Whereas the diagnostic role of IL-6_ascites_ in detection of SBP was already focused on previously, this study offers interesting new aspects. To the best of our knowledge, it is the first one evaluating IL-6_ascites_ in identifying patients without any evidence for infectious diseases. IL-6_ascites_ performed better in “ruling out” apparent infections on admission to ICU than CRP, PCT, or IL-6_serum_. A large study in a total of 368 patients with cirrhosis revealed significantly higher CRP and PCT levels in the case of clinically overt infections [17]. According to a meta-analysis, both CRP and PCT showed acceptable accuracy for diagnosing bacterial infections in patients with liver cirrhosis [45]. It has to be mentioned that none of these studies were carried out in an ICU setting and prevalence of infections was much higher in our population. Infection provokes a release of endogenous mediators involved in inflammatory response with elevated levels in plasma and ascitic fluid [34]. IL-6 is a proinflammatory cytokine typically upregulated in advanced liver disease [15]. As the intraperitoneal accumulation of IL-6 is commonly increased after inflammatory stimuli [46], IL-6_ascites_ seems to be a promising marker of infectious diseases in patients with end-stage liver disease.

Another interesting finding of this study is the potential of IL-6_ascites_ to identify SBP with positive ascitic bacterial culture. Previous studies evaluated the usefulness of PCT to distinguish between “culture-positive SBP” and “culture-negative neutrocytic ascites” (CNNA) [37,47]. According to our results, IL-6_ascites_ was superior to PMN and PCT in discriminative ability. Former reports addressing the prognostic role of culture-negative ascites delivered conflicting results. One study described a lower mortality [48], while another study found a comparable outcome [49]. In the present study, CNNA was not associated with a lower mortality risk compared with “culture-positive SBP”. Anyway, considering the increasing rate of multi-drug resistant bacteria in SBP and the time delay of bacterial cultures, early indices of “culture-positive SBP” based on high IL-6_ascites_ might influence the empirical therapy with broad-spectrum antibiotics.

Renal impairment is a crucial prognostic factor in patients with cirrhosis and occurs in 30–40% of patients with SBP [13,50]. Analogously to previous studies, we found a high correlation of IL-6_ascites_ with creatinine on admission to ICU [25,34]. However, IL-6_ascites_ was a poor predictor of indication for hemodialysis during ICU stay.

Summarizing, the present results suggest a high prognostic relevance of IL-6_ascites_ in critically ill patients with decompensated cirrhosis and acute-on-chronic liver failure. This study reaffirms the diagnostic role of IL-6_ascites_ as a rapidly-available parameter to detect SBP. In addition, it illustrates a promising potential of IL-6_ascites_ to rule out clinically apparent infections and to identify “culture-positive SBP”. Consequently, IL-6_ascites_ offers relevant additional benefits as a supplement to PMN, the “gold standard” in diagnosis of SBP. As analysis of ascitic fluid should be aspired in every patient with end-stage liver disease, IL-6_ascites_ might be a valuable “biomarker” in a challenging population of critically ill patients with liver cirrhosis and ACLF.

### Limitations

Although the results are conclusive with high levels of statistical significance, our retro-prospective study has limitations. It is a single-center study performed exclusively in an ICU setting with a consecutively limited number of patients. Etiology of cirrhosis is mainly alcoholic, whereas NASH-cirrhosis is an increasing burden worldwide and viral-related cirrhosis still plays a major role in developing countries. As a result, subgroup evaluation of IL-6_ascites_ depending on etiology of cirrhosis is limited. Furthermore, patients with cirrhosis are not compared to other populations of critically ill patients. The current study focuses exclusively on baseline assessment and no further measurements of IL-6_ascites_ are available in the course of ICU treatment. Accordingly, the effect of antibiotic regime and intensive care medicine on IL-6_ascites_ is not evaluated. Analysis of IL-6_ascites_ is more costly than assessment of PMN or conventional proinflammatory parameters. Moreover, the availability of measurement is often limited in primary or secondary care compared with large tertiary care centers. Although suggesting a very promising diagnostic and prognostic relevance of IL-6_ascites_, the present evaluation is not sufficient to classify IL-6_ascites_ as a full replacement of PMN. Concerning the detection of SBP in patients with decompensated cirrhosis, IL-6_ascites_ should be regarded as an interesting complement to PMN. Owing to these limitations, further interventional studies with prospective design are needed to more closely specify the potential of IL-6_ascites_ during ICU stay.

## 5. Conclusions

This study illustrates that IL-6_ascites_ is a suitable prognostic “biomarker” to predict outcome in critically ill patients with decompensated cirrhosis and ACLF. In addition to its diagnostic role in detecting SBP, IL-6_ascites_ has a promising potential to identify “culture-positive SBP” as well as to rule out clinically overt infections compared with conventional proinflammatory parameters.

## Figures and Tables

**Table 1:** Patients’ baseline characteristics.

Variables	Result
**Male sex, *n*/total (%)**	43/64 (67%)
**Age, years**	61 (52–67)
**Body weight, kg**	79 (65–87)
**Body height, cm**	175 (167–180)
**BMI**	25.7 (22.9–27.9)
**APACHE II**	23 (17–28)
**SOFA**	10 (8–15)
**MELD**	27 (23–32)
**CTP**	11 (10–12)
**CTP C, *n*/total (%)**	55/64 (86%)
**No ACLF—Grade 0, *n*/total (%)**	9/64 (14%)
**ACLF, *n*/total (%)**	55/64 (86%)
**ACLF Grade I, *n*/total (%)**	16/55 (29%)
**ACLF Grade II, *n*/total (%)**	19/55 (35%)
**ACLF Grade III, *n*/total (%)**	20/55 (36%)
**CLIF-C OF, *n* = 55**	11 (10–12)
**CLIF-C ACLF, *n* = 55**	55 (48–61)
**Etiology of cirrhosis, *n*/total (%)**	Alcoholic 48/64 (75%)
	Viral 4/64 (6%)
	Autoimmune 2/64 (3%)
	Cryptogenic/NASH 10/64 (16%) (histological criteria of NASH 6/10)
**Primary admission diagnoses, *n*/total (%)**	Sepsis 26/64 (41%)
	Acute kidney failure/HRS 18/64 (28%)
	Hepatic encephalopathy 11/64 (17%)
	Gastrointestinal bleeding 9/64 (14%)
**Infection on ICU admission, *n*/total (%)**	53/64 (83%)
**No infection on ICU admission, *n*/total (%)**	11/64 (17%)
**Length of ICU stay, days**	13 (6–24)
**28-days mortality, *n*/total /%)**	28/64 (44%)
**3-month mortality, *n*/total (%)**	39/64 (61%)
**ICU mortality, *n*/total (%)**	34/64 (53%)
**Clinical cause of death, *n*/total (%)**	Sepsis, Pneumonia 30/39 (77%)
	Gastrointestinal bleeding 5/39 (13%)
	Cardiocirculatory failure 4/39 (10%)
**Dialysis during ICU, *n* /total (%)**	36/64 (56%)
**Creatinine, mg/dL**	1.8 (1.4–2.7)
**Bilirubin, mg/dL**	5.4 (2.0–14.9)
**INR**	1.7 (1.5–2.1)
**MAP, mmHg**	73 (68–81)
**Use of vasopressors, *n*/total (%)**	31/64 (48%)
**P_a_O_2_, mmHg**	87 (75–100)
**F_i_O_2_, %**	30 (30–40)
**Mechanical ventilation, *n*/total (%)**	24/64 (38%)
**HE, *n*/total (%)**	34/64 (53%)
**WBC, 10^9^ cells/L**	10.7 (8.0–13.6)

BMI: body mass index; APACHE: acute physiology and chronic health evaluation II; SOFA: sequential organ failure assessment; MELD: model of end-stage liver disease; CTP: Child–Turcotte–Pugh; ACLF: acute on chronic liver failure; CLIF-OF: chronic liver failure organ failure score; CLIF-ACLF: chronic liver failure acute on chronic liver failure score; NASH: non-alcoholic steatohepatitis; HRS: hepato-renal syndrome; INR: international normalized ratio; MAP: mean arterial pressure; P_a_O_2_: arterial partial pressure of oxygen; F_i_O_2_: fraction of inspired oxygen; HE: hepatic encephalopathy; WBC: white blood cell count.

**Table 2:** Percentage distribution depending on evidence of infection and corresponding levels of ascitic interleukin 6 (IL-6_ascites_) on admission to intensive care unit (ICU).

Classification		Percentage (Fraction)	IL-6_ascites_, pg/mL	*p*-Value
	***No infection***	17% (11/64)	1031 (694–1713)	<0.001
	***Infection***	83% (53/64)	8607 (4282–25,249)	
	***No SBP***	70% (45/64)	4275 (1169–8526)	<0.001
	***SBP***	30% (19/64)	24,453 (12,329–63,836)	
	***Community-acquired SBP***	32% (6/19)	17,159 (10,703–39,539)	0.323
	***Nosocomial SBP***	68% (13/19)	38,679 (11,425–78,809)	
	***CNNA***	47% (9/19)	12,528 (6321–21,198)	0.008
	***Culture-positive SBP***	53% (10/19)	61,155 (28,003–84,643)	
**Single-infections**	**Urinary tract**	11% (7/53)	2107 (1453–4123)	
	**Respiratory**	30% (19/53)	7045 (2149–9419)	
	**SBP**	11% (7/53)	33,228 (12,329–63,839)	
	**Bacteremia**	5% (3/3)	9624 (1725–10,558)	
**Co-** **infections**	**Pneumonia + Urinary tract**	5% (3/53)	6690 (4289–16,958)	
	**Pneumonia + SBP**	14% (9/53)	16,375 (11,425–77,525)	
	**Urinary tract + SBP**	6% (4/53)	34,375 (8497–73,592)	
	**Pneumonia + Bacteremia**	1% (1/53)	9591	

ICU: intensive care unit; IL-6_ascites_: ascitic interleukin 6; SBP: spontaneous bacterial peritonitis; CNNA: culture-negative neutrocytic ascites.

**Table 3:** Correlations and linear regressions of baseline ascitic interleukin 6 (IL-6_ascites_) with laboratory parameters and clinical scores on admission to ICU.

	Spearmans Coefficient r_s_	Linear Regression R^2^	*p*-Value
**CRP, mg/dL**	0.453	0.055	<0.001
**PCT, ng/mL**	0.445	0.019	<0.001
**IL-6_serum_, pg/mL**	0.658	0.150	<0.001
**APACHE-II**	0.494	0.236	<0.001
**SOFA**	0.570	0.281	<0.001
**MELD**	0.434	0.287	<0.001
**CTP**	0.160	0.167	0.207
**ACLF-Grade**	0.375	0.108	0.002
**CLIF-OF**	0.330	0.234	0.014
**CLIF-ACLF**	0.381	0.202	0.004

ICU: intensive care unit; IL-6_ascites_: ascitic interleukin 6; CRP: C-reactive protein; PCT: procalcitonin; IL-6_serum_: serum interleukin 6; WBC: white blood cell count; APACHE: acute physiology and chronic health evaluation; SOFA: sequential organ failure assessment; MELD: model of end-stage liver disease; CTP: Child–Turcotte–Pugh; ACLF: acute on chronic liver failure; CLIF-OF: CLIF organ failure score; CLIF-ACLF: CLIF acute-on-chronic liver failure score.

## Supplementary Materials

The following are available online at https://www.mdpi.com/2077-0383/9/9/2865/s1. Figure S1: Correlation of ascitic interleukin 6 (IL-6_ascites_) with ascitic polymorphonuclear neutrophils (PMN) in patients with spontaneous bacterial peritonitis (“*SBP*”, *n* = 19). Figure S2: Correlation of ascitic interleukin 6 (IL-6_ascites_) ascitic with baseline creatinine. Figure S3: Predictive value of baseline ascitic interleukin 6 (IL-6_ascites_) compared with APACHE-II, SOFA, MELD, CTP, and ACLF-grade in identifying patients with need for hemodialysis therapy during ICU stay.
