# Supplementary material for: Ascitic Interleukin 6 Is Associated with Poor Outcome and Spontaneous Bacterial Peritonitis: A Validation in Critically Ill Patients with Decompensated Cirrhosis
Source: J Clin Med. 2020 Sep 4;9(9):2865. doi: 10.3390/jcm9092865 (PMC7564827; doi:10.3390/jcm9092865)

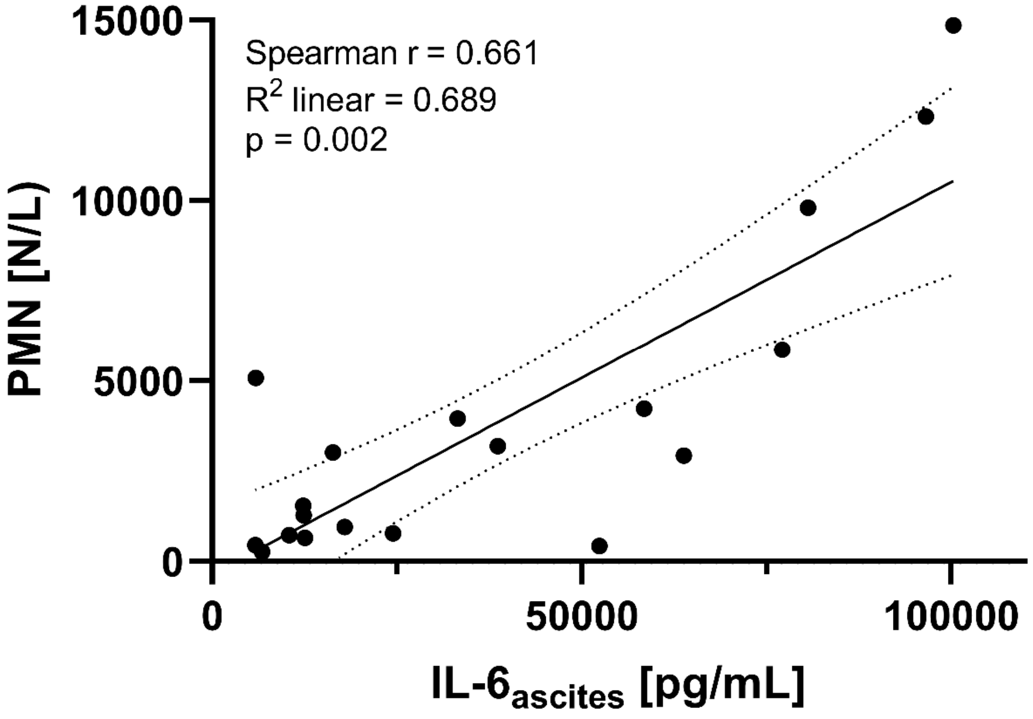

Figure 1. Correlation of ascitic Interleukin 6 ($IL\text{-}6_{ascites}$) with ascitic polymorphonuclear neutrophils (PMN) in patients with spontaneous bacterial peritonitis ("SBP", *n* = 19).

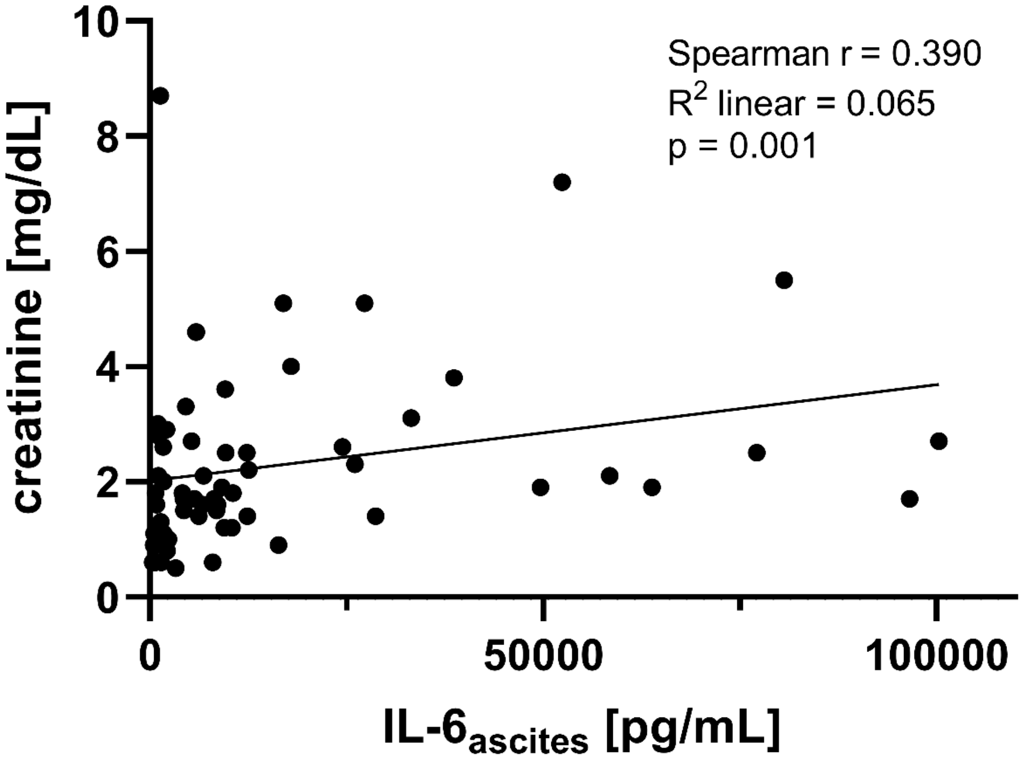

Figure S2: Correlation of ascitic Interleukin 6 (IL-$6_{ascites}$) ascitic with baseline creatinine

Figure S3: Predictive value of baseline ascitic Interleukin 6 ($IL\text{-}6_{ascites}$) compared to APACHE-II, SOFA, MELD, CTP and ACLF-Grade in identifying patients with need for hemodialysis therapy during ICU-stay

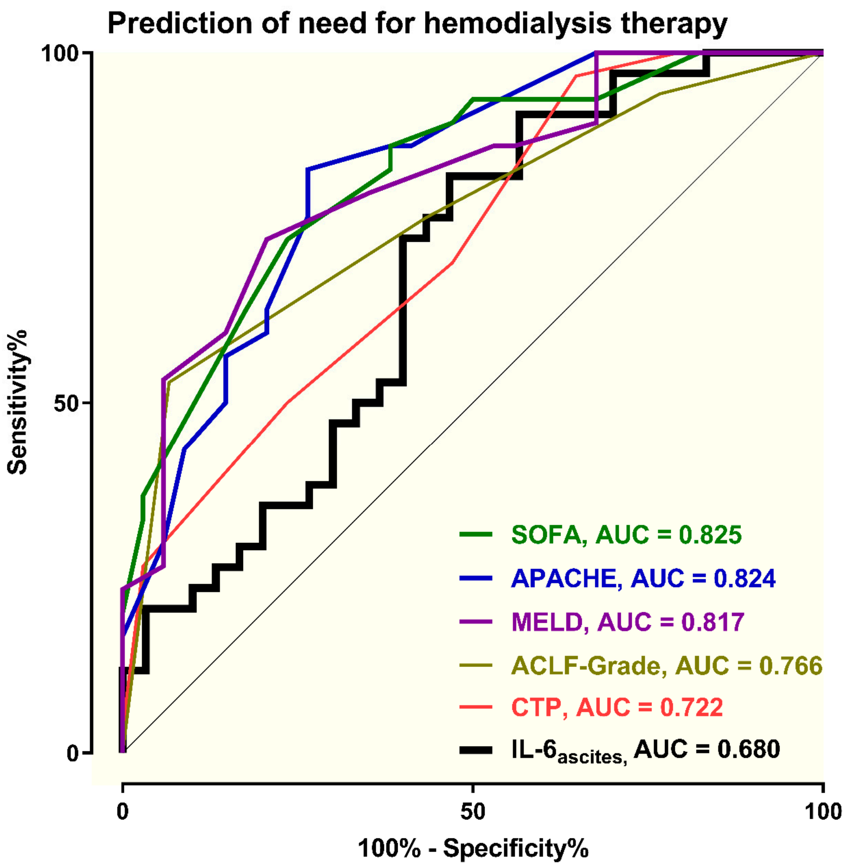

Supplement: Supplementary file 1 [file jcm-09-02865-s001.pdf]
